# The Value of the Follow-Through Derives from Motor Learning Depending on Future Actions

**DOI:** 10.1016/j.cub.2014.12.037

**Published:** 2015-02-02

**Authors:** Ian S. Howard, Daniel M. Wolpert, David W. Franklin

**Affiliations:** 1 Centre for Robotics and Neural Systems, University of Plymouth, Portland Square, Plymouth PL4 8AA UK; 2 Computational and Biological Learning Lab, Department of Engineering, University of Cambridge, Trumpington Street, Cambridge CB2 1PZ, UK

## Abstract

In ball sports, we are taught to follow through, despite the inability of events after contact or release to influence the outcome [1, 2]. Here we show that the specific motor memory active at any given moment critically depends on the movement that will be made in the near future. We demonstrate that associating a different follow-through movement with two motor skills that normally interfere [3–7] allows them to be learned simultaneously, suggesting that distinct future actions activate separate motor memories. This implies that when learning a skill, a variable follow-through would activate multiple motor memories across practice, whereas a consistent follow-through would activate a single motor memory, resulting in faster learning. We confirm this prediction and show that such follow-through effects influence adaptation over time periods associated with real-world skill learning. Overall, our results indicate that movements made in the immediate future influence the current active motor memory. This suggests that there is a critical time period both before [8] and after the current movement that determines motor memory activation and controls learning.

## Highlights

• Future movements determine which motor memory is currently active and modifiable
• Skills that otherwise interfere can be learned if each has a unique follow-through
• A single skill is learned faster if its follow-through is consistent
• Selection of motor memories depends on both lead-in and follow-through movements

## Teaser

For a motor skill to be learned, its motor memory must be stored, protected from interference, and reactivated for modification during practice. Howard et al. demonstrate that future movement determines which motor memory is currently active and that a consistent follow-through constrains skill acquisition to a single memory, thereby speeding up learning.

## Results and Discussion

For a motor skill to be learned over a prolonged period of time, the motor memory of the skill must be stored, protected from interference by intervening tasks, and reactivated for modification when the skill is practiced. Given the widespread notion of the importance of a consistent follow-through in many sports [1, 2], here we examine whether the currently active motor memory might depend on the movement that we are going to make in the near future. We examine a motor skill that is known to be long lasting but also subject to interference—learning to reach in the presence of a dynamic (force-field) perturbation generated on the hand by a robotic interface [4, 9]. When two force fields that act in opposing directions are presented alternately, there is substantial interference, preventing learning of either [4–7]. We first examined whether linking such skills that interfere to different follow-through movements might activate separate motor memories for each, thereby allowing both skills to be learned without interference.

**Figure 1:**
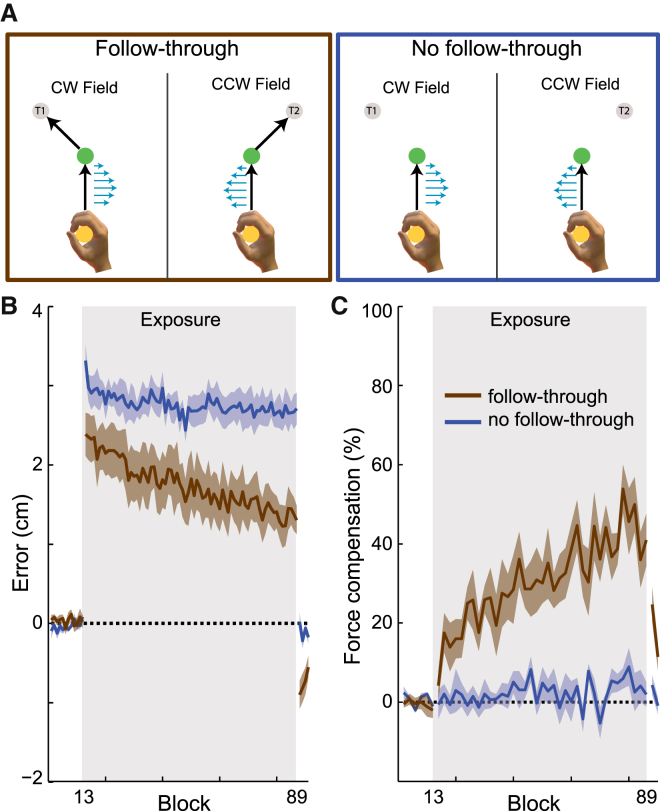
Associating Different Follow-Through Movements with Motor Skills Reduces Interference (A) Participants made an initial movement to a central target (green circle). During exposure trials, a velocity-dependent curl force field (force vectors shown as blue arrows) was applied during this movement, and the field direction (clockwise [CW] or counter-clockwise [CCW]) was determined by a visual target location (T1 or T2). A follow-through group made a subsequent unperturbed movement to the target location, whereas a no-follow-through group remained at the central target. The directions of the force-field (CW or CCW) and follow-through movement (+45° or −45°) were counter-balanced across participants. Participants made movement in four directions but for clarity only one direction is shown. (B and C) The kinematic error (B) and force adaptation (C) (mean ± SE across participants for pairs of blocks, combining adjacent even and odd blocks) for the follow-through (brown) and no-follow-through (blue) groups. See also Figures S1 and S2 and Table S1.

Participants grasped the handle of a robotic interface (Figure S1) and made a reaching movement (in one of four directions) through a perturbing force field to a central target, followed immediately by a second unperturbed, follow-through movement to one of two possible final targets (Figure 1A, follow-through; see the Supplemental Experimental Procedures for full details). The field direction (clockwise or counter-clockwise) was randomly selected on each trial but was uniquely specified by the target (present throughout the trial) to which the follow-through movement would be made (association of force-field direction and follow-through movement was counter-balanced across participants). So that predictive force compensation could be assessed independently from co-contraction, channel trials [10, 11], in which the movement was confined to a simulated mechanical channel from the starting to central target, were randomly applied throughout the experiment. Participants performed 75 blocks of 18 trials each in the force field, and to examine learning, we compared differences in the kinematic error and force compensation between the first four blocks and final four blocks in the exposure phase using an ANOVA with a main factor of epoch (two levels) and random factor of participant (eight levels). We found both a significant reduction in kinematic error (Figure 1B, brown; F_1,7_ = 16.8; p = 0.005; hand paths shown in Figure S2A) and increase in force compensation (Figure 1C, brown; F_1,7_ = 17.706; p = 0.004) reaching around 40% of full compensation over a session. In contrast, when a second group of participants were presented with the final target, which again was predictive of the field direction, but did not follow-through to the target (Figure 1A, no follow-through), there was substantial interference between the motor skills, as expected [8, 12, 13]. Although we observed a small reduction in kinematic error in this group (Figure 1B, blue; F_1,7_ = 12.371; p = 0.01; hand paths shown in Figure S2B) there was no significant increase in force compensation (Figure 1C, blue; F_1,7_ = 0.434; p = 0.531), suggesting that participants solely used non-specific co-contraction to reduce their error [14–16]. Finally, we contrasted the adaptation in the two groups of subjects using an ANOVA with epoch (two levels) and group (follow-through or no follow-through). There was a significant interaction effect for both kinematic error (F_1,124_ = 7.388; p = 0.08) and force compensation (F_1,124_ = 21.55; p < 0.001), indicating that interference was strongly reduced in the follow-through group.

These results show that (despite the unperturbed kinematics of the movements to the central target being similar for both final targets; Table S1), when a follow-through movement is made that is predictive of the field direction, there is substantial reduction in interference. This suggests that different follow-throughs may activate distinct motor memories. Therefore, during skill learning on a single task, identical future movements on each trial (i.e., consistent follow-through) may access a single motor memory. In contrast, a variable follow-through may access multiple motor memories across trials, with any learning being spread across multiple memories, leading to a decrease in the speed of skill acquisition.

**Figure 2:**
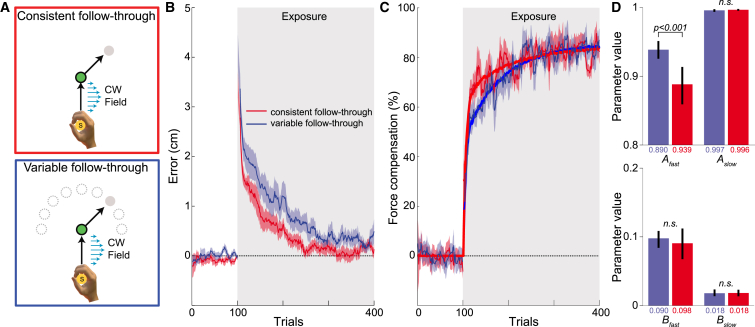
Consistent Follow-Through Improves Learning Rate (A) Participants made a movement to a central target (green circle) followed by a follow-through movement to a target. During exposure trials, a curl force field was applied on the movement to the central target. The consistent-follow-through group always made the follow-through movement to the same target, whereas for the variable-follow-through group the target was randomly selected from nine possible locations on each trial. The direction of the force-field and follow-through movement was counter-balanced across participants. (B and C) The kinematic error (B) and force adaptation (C) (ten-trial running mean ± SE across participants) for consistent-follow-through (red) and variable-follow-through (blue) groups. Solid lines show fits of a dual-rate model to force compensation. There are 40 channel trials for each participant, plotted according to the trial number at which they were presented in a pseudo-random fashion. (D) Parameters of fits (with 95% confidence intervals) of the dual-rate model to both groups. See also Table S2.

We tested this prediction in two groups who experienced a single force field whose direction was fixed across all trials. Both groups made a movement in the force field to a central target followed by an unperturbed follow-through movement to a final target (Figure 2A). For one group, the follow-through movement was always made to the same target (consistent follow-through), whereas the other group made a follow-through movement on each trial to a randomly selected target from nine possible locations (variable follow-through). The direction of the force-field and follow-through movements was counter-balanced across participants. Importantly, the kinematics (and variability) of unperturbed movements to the central target were not significantly different between the two groups (Table S2), and there was no difference in the initial errors in the force field between the two groups (ANOVA on maximum perpendicular error of the first two exposure trials with a main factor of experimental group: F_1,46_ = 0.801; p = 0.375). However, significantly faster learning was observed for the consistent, compared to the variable, follow-through group for both kinematic error (Figure 2B; F_1,5940_ = 155.041; p < 0.001) and force compensation (Figure 2C; F_1,660_ = 3.921; p = 0.048) as shown using an ANOVA across all exposure trials with the main effects of experimental group and trial number. The same analysis, performed on the first one-third of the exposure trials, showed significantly faster learning for the consistent compared to the variable follow-through group for both kinematic error (F_1,1980_ = 93.171; p < 0.001) and force compensation (F_1,220_ = 9.057; p = 0.003). By the end of the session, there were no significant differences in either the kinematic error (F_1,94_ = 1.668; p = 0.2) or force compensation (F_1,94_ = 0.163; p = 0.687) (ANOVA on the last four trials with the main effect of experimental group), showing that both groups eventually learned the same amount for this simple skill.

We used a dual-rate model to examine whether changes in the parameters that govern learning might account for the differences that we observed in skill acquisition rate, but not final level of learning. The time course of learning novel dynamics is well accounted for by two interacting processes: a fast process that adapts and decays quickly and a slower process that adapts and decays more gradually [17]. Each process is characterized by a learning rate that controls how strongly the motor memory is updated based on errors and a retention factor determining the movement-to-movement retention of the motor memory. We fit this dual-rate model to our participants’ learning (model is fit to the group-averaged data; Figure 2C, thick lines), and this showed that the differences between the groups was primarily due to the retention factor of the fast process (Figure 2D; A_fast_ p < 0.001 between groups, other parameters non-significant), suggesting that variable follow-through leads to decreased retention across trials [18].

Could our results on simple force-field learning over the course of an experimental session apply to real-world learning taking place over much more extended periods? In real-world tasks, such as a tennis or golf stroke, the lead-in to the movement is critical for task success, as it will determine characteristics such as variability at contact [19, 20]. Moreover, the recent past has been shown to also affect the selection of the current motor memory [8]. We examined the extent to which two motor skills, opposing force fields, could be simultaneously learned when the skill being currently experienced depended on a nonlinear combination of the past (lead-in) and future (follow-through) movements.

**Figure 3:**
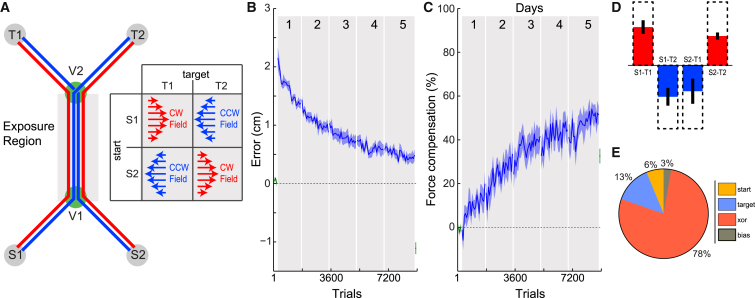
Participants Show Extended Learning of Skills that Nonlineary Depend on Both Lead-In and Follow-Through (A) Participants made movements from one of two start locations to one of two target locations (four possible movements). Movements had to pass through two via points (V1 and V2). On each exposure trial, a force field was applied in the region between the via points (exposure region), and the field direction (CW or CCW) was uniquely specified by the combination of the start and target locations according to an XOR rule (table). The direction of the force field (CW or CCW) was counter-balanced across participants. (B and C) The kinematic error (B) and force adaptation (C) (mean ± SE across participants for pairs of blocks, combining adjacent even and odd blocks) over the 5 days of the experiment. (D) The force compensation averaged over the last 20 blocks (mean ± SE across participants) for each of the four movements, with dashed bars indicating full compensation. (E) Average of the variance explained by a regression analysis across participants. The regression analysis was used to predict the pattern of compensation (D) by fitting weights to the four possible patterns (bias, start, target, XOR) across the movements. The percentage shows the amount of the variance in force compensation explained by these four patterns (see the Supplemental Experimental Procedures for details). See also Figure S3.

Participants made movements from two possible starting locations (Figure 3A; S1 or S2) through two via points (V1 and V2) to one of two possible target locations (T1 or T2). A force field was applied between the via points whose direction on each trial was uniquely specified, according to an exclusive-or (XOR) rule, by the starting and target locations used on that trial (Figure 3A). Critically, the direction of the force field could not be predicted based on either the start location or the target location alone, but rather depended on both the start and final locations in a non-linear manner. The direction of the force field relative to the movements was counter-balanced across participants. Participants performed 240 blocks of 26 trials over 5 days, and to examine learning, we compared differences in the kinematic error and force compensation for the first four blocks and final four blocks in the force field using an ANOVA with a main factor of epoch (two levels) and random factor of participants (six levels). This was a surprisingly hard task to learn, and over the 5 days of practice, participants showed both a strong and gradual reduction in error (Figure 3B; F_1,5_ = 36.750; p = 0.002) and increase in force compensation (Figure 3C; F_1,5_ = 61.981; p = 0.001) to around 50%. Participants learned to access the motor memories based on the nonlinear rule as shown by the force compensation being of the appropriate sign for all four possible movements (Figures 3D and S3), with the largest variance in the data accounted for by an XOR rule (Figure 3E). These results demonstrate that participants can utilize both past and future movements to produce a nonlinear separation of motor memories. This shows that the temporal events in close proximity to a movement, both before and after, are critical in determining the motor memory in which the skill is stored. Moreover, even for a relatively simple skill, such as force-field learning (compared to a tennis or golf stroke), the learning is slowly acquired over time courses on the order of real-world skill learning and is highly dependent on both the lead-in and follow-through. Although we have focused on learning simple force fields in constrained arm movements, previous work suggests that the mechanisms underlying such learning generalize to whole-body movement such as posture [21] and walking [22–24], as well as other more naturalistic movements [25].

Previous studies have examined a range of contextual cues that might allow the separation of motor memories. While static cues (e.g., color) have a very limited ability to separate motor memories [12, 13], dynamic moving cues [13, 26], different concurrent motion of the other arm [27–29], or the lead-in to a movement [8, 30] often have a substantial effect. Moreover, separating the location of learning either proprioceptively or visually facilitates learning of opposing force fields [13, 31, 32]. Models of such contextual effects in the motor system posit that they arise from the engagement of separate neural populations, e.g., [27]. Since it known that that future motor planning affects neural activity [33], it seems likely that the follow-through effect that we report either directly engages the separate neural populations that leads to the generation of movement or does so by affecting the initial state of the dynamical systems of neurons in the motor system that control movement [34].

Although we have shown that consistent follow-through leads to faster learning through selection of a single memory, this does not preclude other potential advantages of the follow-though, such as injury reduction or other biomechanical advantages [2]. Although several contextual cues have previously been shown to reduce interference [13, 26, 27, 29, 30, 32, 35, 36], our study is the first to show that different follow-through movements can reduce interference substantially, demonstrating the importance of future motor events in controlling current motor learning. Our findings suggest that distinct follow-throughs associated with different motor skills, such as different tennis strokes, will help maintain these skills in separate motor memories, thereby protecting them from interference when learning other skills. Moreover, even for a single skill, maintaining a consistent follow-through will speed up learning. An intriguing question is why a particular follow-through might be preferred when learning a skill. Our results suggest that variability in the follow-through, which might arise from planning variability [37], motor noise [19, 20, 38], or other sources of variability [39], would lead to a reduction in the speed of skill acquisition. Therefore, it may be optimal to choose the follow-through for a skill that can be executed with the minimum variability.

## Author Contributions

I.S.H., D.M.W., and D.W.F. designed the study. I.S.H. ran the experiments. I.S.H., D.M.W., and D.W.F. performed the data analysis. I.S.H., D.M.W., and D.W.F. wrote the paper.

## Supplemental Information

Document S1. Supplemental Experimental Procedures, Figures S1–S3, and Tables S1 and S2

Document S2. Article plus Supplemental Information
