# Supplementary material for: The Value of the Follow-Through Derives from Motor Learning Depending on Future Actions
Source: Curr Biol. 2015 Feb 2;25(3):397–401. doi: 10.1016/j.cub.2014.12.037 (PMC4320013; doi:10.1016/j.cub.2014.12.037)
Supplement: Document S1. Supplemental Experimental Procedures, Figures S1–S3, and Tables S1 and S2 [file mmc1.pdf]

Current Biology

Supplemental Information

The Value of the Follow-Through Derives from Motor Learning Depending on Future Actions

Ian S. Howard, Daniel M. Wolpert, and David W. Franklin

Supplemental Figures

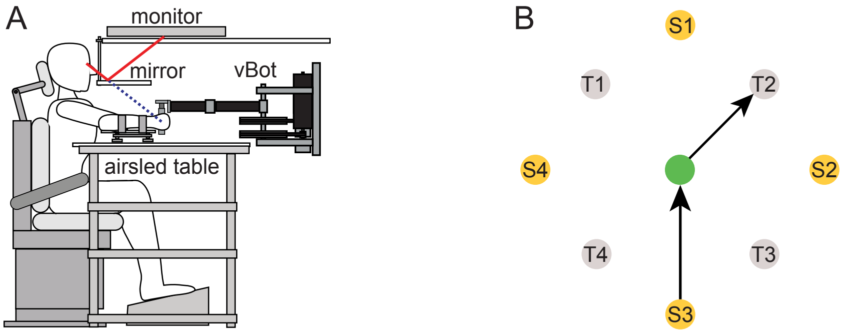

Figure S1. Experimental Paradigm.

(A) The participant grasps the handle of the robotic manipulandum (vBOT) while seated. Visual feedback of movements is presented veridically using a horizontally mounted monitor viewed through a mirror. The participant's forearm is fixed to the handle and supported by an airsled.

(B) Workspace layout for Experiment 1. There were four possible final target locations (grey circles: T1-T4), one central target (green circle, note that in the experiment this was displayed as grey) and four start target locations (yellow circles: S1-S4). For each start location, two possible final target locations could be chosen (one for each force-field direction) corresponding to ±45° angle relative to the initial movement direction. The arrows show an example of the movements in a single trial for the follow-through group.

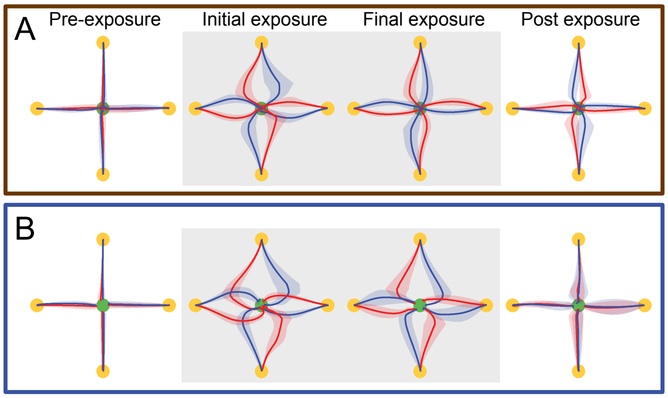

Figure S2. Hand paths for the four different movements to the central target for Experiment 1.

(A) Hand paths (mean ± s.d. across participants) to the central target during different phases on the experiment for the follow-through group; pre-exposure (last block), initial exposure (first block), final exposure (last block), post exposure (first block). Each trajectory is the average of the first trajectory from each participant within each relevant block.

(B) Hand paths to the central target for the no follow-through group.

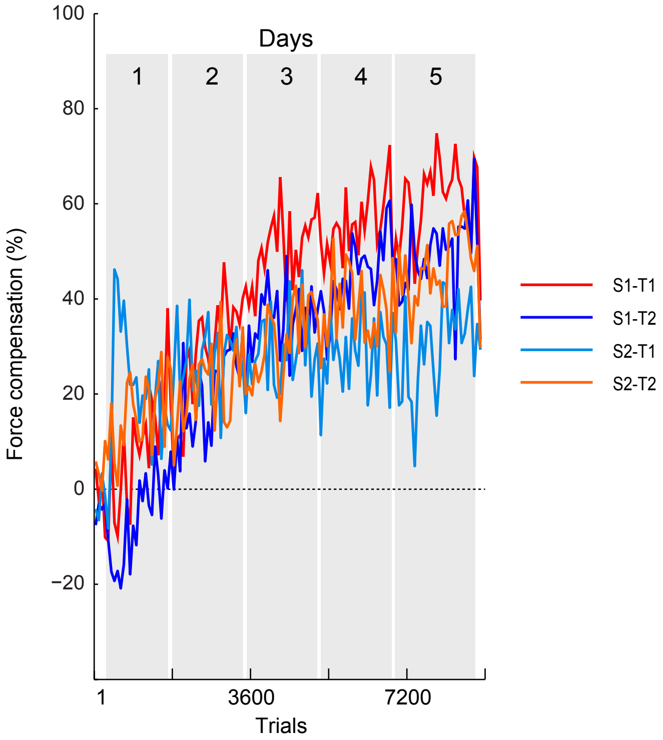

Figure S3. Evolution of force compensation for each movement in Experiment 3.

The force adaptation (mean across participants for pairs of blocks) over the 5 days of the experiment for each of the four movements.

Supplemental Tables

	No follow-through group				Follow-through group				Group difference		
	Target				Target						
Measure	Left	Right	$F_{1,7}$	p	Left	Right	$F_{1,7}$	p	Difference	$F_{1,14}$	p
Lateral deviation (cm)	-0.02	-0.04	0.41	0.542	0.01	-0.08	5.88	0.046	0.00	0.000	0.988
Path length (cm)	9.7	9.8	1.87	0.214	9.7	9.7	0.45	0.522	-0.03	0.65347	0.432
Duration (s)	0.272	0.273	0.04	0.852	0.235	0.237	0.84	0.390	-0.036	6.3768	0.024
Peak speed (cm/s)	46.0	46.1	0.07	0.796	52.9	52.7	0.66	0.444	6.7	4.0337	0.064
Central target speed (cm/s)	27.4	27.2	0.24	0.642	33.5	32.9	2.10	0.191	5.9	5.8287	0.030
Dwell time (s)					0.146	0.151	1.45	0.268			

Table S1. Analysis of the kinematics for the pre-exposure (null field) trials of Experiment 1.

For each group we performed a repeated measures ANOVA for each kinematic measure as a factor of final target direction (2 levels). Table shows means for each group, the F statistics and p-values comparing left and right final targets (i.e. different follow-troughs or visual targets). We also examined the differences between the groups using a repeated measures ANOVA with a single factor of group. The table shows the mean difference between the groups, F statistics and p values. Note that, with one exception, the differences between the two targets are not significant. The only significant difference is the lateral deviation at mid-movement for which the mean difference is 0.9 mm and below the spatial difference that allows opposing fields to be learned [S1, S2]. Not surprisingly, there are differences in the kinematic parameters and duration and central target velocity for the follow-through compared to no follow-through groups, as the latter were required to stop at the central target. Other kinematic parameters were not significantly different.

	Kinematic measures				Kinematic variability (SD)			
	Group		Difference		Group		Difference	
**Measure**	**Consistent**	**Variable**	**$F_{1,22}$**	**p**	**Consistent**	**Variable**	**$F_{1,22}$**	**p**
Lateral deviation (cm)	-0.019	-0.095	0.81	0.38	0.41	0.44	0.35	0.56
Path length (cm)	15.7	15.7	0.89	0.35	0.45	0.41	0.07	0.79
Duration (s)	0.34	0.34	0.26	0.62	0.077	0.084	0.04	0.84
Peak speed (cm/s)	63.3	64.7	0.11	0.74	8.1	8.3	0.03	0.86
Central target speed (cm/s)	28.6	28.2	0.06	0.81	10.5	10.6	0.01	0.92
Dwell time (s)	0.291	0.269	1.27	0.27	0.079	0.074	0.18	0.68

Table S2. Analysis of the kinematics for the pre-exposure (null field) trials of Experiment 2.

For each kinematic measure we examined whether the two groups (variable and consistent follow-through) differed in their mean values and in their variability (as reflected in the SD of the kinematic measured calculated within each participant). We used repeated measure ANOVAs with a single factor of group. The table shows the mean and the average SD for each group and the F statistics and p values testing for the difference between the groups. All tests showed the differences failed to reach significance ($p>0.27$ for all).

Supplemental Experimental Procedures

A total of 46 right-handed participants (16 female; age 22.7±4.9 mean±sd years) took part in the experiments. Participants provided written informed consent and were naïve to the aims of the experiments. Local ethics committees in Cambridge and in Plymouth approved the protocol and all participants were right handed based on the Edinburgh handedness questionnaire [S3].

Apparatus

Experiments were performed using a vBOT planar robotic manipulandum, with associated virtual reality system and air table (for details see [S4]). Participants were seated in a sturdy chair in front of the apparatus and firmly strapped against the backrest with a four-point seatbelt to reduce body movement. Participants grasped the robot handle in their right hand while an air sled (constraining movement to the horizontal plane) supported their right forearm. Handle position and endpoint force (Nano 25 6-axis transducer; ATI) were recorded at 1000 Hz for offline analysis using Matlab (Matlab, The MathWorks Inc., Natick, MA, USA).

Visual feedback was provided using a computer monitor mounted above the vBOT and was projected veridically to the participant via a mirror. The virtual reality system was used to overlay images such as targets (1.25 cm radius disks) and a hand cursor (0.5 cm radius red disk) in the plane of movement.

Experiment 1 – The effect of follow-through movements on interference (n=*16*).

Experiment 1 was designed to examine the ability of future movements to separate current motor memories. On each trial a starting, central and final target was displayed. The central target was in a fixed position on all trials, approximately 30 cm below the eyes and 30 cm in front of the chest. There were four possible starting targets positioned 12 cm from the central target and arranged at 0°, 90°, 180° and 270°.

The final target was 10 cm from the central target and located either at -45° or +45° relative to the starting-to-central target direction (Fig. S1).

At the beginning of a trial, the starting location was displayed to which the vBOT moved the participant's hand (following a minimum jerk trajectory), at which point the central and a final target were displayed. Participants were required to remain within the start target for 300 ms, after which they were cued by a tone to initiate the movement. Participants were required to make a movement to the central target during which either a null field, a velocity-dependent curl force-field [S5] or a force channel was presented. In null field trials, the vBOT generated no force. In the curl force-field trials, the force generated by the vBOT was given by:

$$\begin{bmatrix} F_x \\ F_y \end{bmatrix} = k \begin{bmatrix} 0 & -1 \\ 1 & 0 \end{bmatrix} \begin{bmatrix} \dot{x} \\ \dot{y} \end{bmatrix}$$

where *k* was set equal to ±13 N m^{-1} s. The sign of *k* determined the direction of the force-field (clockwise CW or counterclockwise CCW). The direction of the force-field was determined by the whether the final target was displayed at +45° or -45° relative to the movement to the central target (e.g. +45=CW & -45°=CCW). The assignment between direction to the final target (+45/-45°) and curl field direction (CW/CCW) was fixed within a participant but counterbalanced across participants.

Channel trials were used to assess feedforward adaptation. In a channel trial, the movement was confined to a simulated mechanical channel from the starting to central target with a spring constant of 10,000 N/m and a damping coefficient of 50 $Nm^{-1}s$ orthogonal to the wall [S6, S7].

A block consisted of 16 field trials and 2 channel trials. In the field trials, each of the 4 starting targets and 2 possible targets was repeated twice. The order of the trials within a block was pseudo-random, except that a channel trial always occurred within the first and last four trials of each block. Channel trials were

only applied for movements from the 0° start position, with one trial for each of the two possible final targets (randomizing which came first within each block).

Each experiment began with a pre-exposure phase consisting of 12 blocks in which no forces were applied (216 null trials), followed by an exposure phase of 75 blocks (1350 field trials), and finally a post-exposure phase consisting of 4 blocks (72 null trials). Participants were given a short rest on average every 200 trials (195-205 trials).

Participants were allocated to one of two groups: a no follow-through (n=8) and a follow-through group (n=8), that differed only in events after they had reached through the force-field to the central target. The no follow-through group was required to stop within the central target after which the trial ended. The follow-through group was required to continue the movement from the central target to the final target and they were not required to stop at the central target. If they did not pass through the central target, the trial was terminated. Forces generated by the vBOT were turned off after participants entered the central target, so that movements from the central target to the final target were always made in a null field.

After each trial, participants were given feedback on the duration of their movement to the central target. This was the time from when the hand exceeded a speed of 30 cm/s after it had left the start target until it entered the central target (no follow-through group) or passed the middle of the central target (follow-through group). If the duration was between 150-250 ms, a "correct speed" message was displayed, otherwise a too fast / too slow warning was given. Participants were required to repeat a trial if they failed to achieve a speed greater than 30 cm/s in the movement to the central target.

Analysis

The kinematic error was calculated on each movement to the central target as the maximum perpendicular error (MPE) of the hand path relative to a straight line joining the movement's start and the central target. For each participant, the MPE for all trials was averaged within a block, with the sign

appropriately reversed so that errors from CW and CCW field trials could be combined. The MPE mean and standard error (SE) was computed across all participants. For plotting, the mean and SE was calculated for each adjacent pair of blocks, combining odd and even blocks (blocks 1 & 2, 3 & 4 etc).

To assess feedforward learning independent of co-contraction we analyzed the force produced on channel trials [S7, S8]. The force produced by participants orthogonal to the channel wall was summed across the movement to the central target. This value was expressed as a percentage adaptation (termed Force Compensation) by calculating the value that would perfectly compensate for the field, as determined by the field strength and the summed movement velocity on that trial [S1, S9].

We also examined whether the kinematics of the movements to the central target depended on whether the final target was at -45° (leftward) or +45° (rightward) of the initial movement. If unperturbed movements to the central target are substantially different for the two possible final targets this could facilitate learning [S1, S2]. We therefore examined the pre-exposure trials (null field trials) as these provide a fair comparison as we expect substantial differences during field trials (as the field direction are different for the different final targets). We calculated 6 kinematic measures for each movement to the central target. We selected the part of the movement from when the hand left the starting location to when it entered the central target and calculated the duration, path length and peak speed of this movement, as well as the final speed as the hand entered the central target. We also calculated the signed lateral deviation from the straight line joining the starting and central target when the hand was midway to the central target. In addition, for the follow-through group we calculated the dwell time that the hand spent within the central target. Within each group we compared these measures for the leftward and rightward targets and also compared these measures across the two groups (collapsed across targets). For each group we performed a repeated measures ANOVA for each kinematic measure as a factor of final target direction (2 levels). Here, multiple ANOVAs are more appropriate than a single MANOVA, as we wish to reduce the chances of a type II error.

We performed hypothesis-based planned comparisons and report uncorrected p-values to determine statistical significance. Statistical differences were determined using an ANOVA in SPSS using the general linear model. Differences in kinematic error or force compensation between the first 4 blocks and final 4 blocks in the exposure phase were examined using an ANOVA with a main factor of epoch (2 levels) and random factor of participants (8 levels). To contrast the adaptation in the two groups an ANOVA was performed with main factors of epoch (2 levels) and experimental group (2 levels: follow-through and no follow-through) to examine whether the interaction between epoch and group was significantly different. Statistical significance was considered at the $p < 0.05$ level for all statistical tests.

Experiment 2 – The effect of follow-through variability on learning (n=24).

We examined how follow-though variability affected the rate of learning of a single force-field. The experiment was similar to first experiment, except that only a single starting location (0°) and single force-field (field constant k increased to 16 N m^{-1} s) was experienced by each participant (Fig. 2A). The field direction (CW or CCW) was counterbalanced across participants, but each participant only experienced one force-field.

Participants were allocated to one of two groups: a consistent follow-through (n=12) and a variable follow-through group (n=12) that differed only in the movement they were required to make after they had reached through the field to the central target (located 18 cm from the start location). The consistent follow-through group always reached to the same final target (counterbalanced at +45° or -45° across participants) whereas for the variable follow-through group the final target was selected on each from one of 9 possible locations. The set of possible final targets were 10 cm from the central target, and arranged between -90° to +90° in 22.5° steps (Fig. 2A). To allow comparison with the consistent follow-through group, for half the participants channel trials occurred only for the +45° final target and for the other half for the -45° target.

Participants were encouraged to pause briefly at the central target on the way to the final target. If they remained within the central target for less than 50 ms a warning was given at the end of the trial. This small pause within the central target was required so as to maintain similar durations and speeds for the movement to the central target for all follow-through directions. The duration of the dwell time was kept small (50 ms) as the strength of the follow-through effect is likely to decay as dwell time increases [S9]. Movement duration was calculated as the time between when the hand left the starting location until it was within the central target. If the duration was within 200-300ms, "great" was displayed, otherwise if within 150-350ms, a "good" was displayed. Outside this range, a too fast / too slow warning was given.

A block consisted of 9 null/field trials and 1 channel trial. Channel trials used a spring constant of 6,000 N/m and a damping field constant of 30 Nm^{-1} s. Each experiment began with a pre-exposure phase consisting of 10 blocks in which no forces were applied (100 null field trials), followed by an exposure phase of 30 blocks (300 field trials), and finally a post-exposure phase consisting of 10 block (100 null trials). There were 40 channel trials in total (10 in the pre-exposure and 30 during the exposure phase).

Analysis

Initial differences in kinematic error upon introduction of the force-field was examined using the MPE on the first 2 exposure trials with an ANOVA with a main factor of experimental group (2 levels). The speed of learning was examined for both kinematic error and force compensation using an ANOVA across all of the exposure trials and over the first third of the exposure trials. The ANOVA was performed with a main effect of experimental group (2 levels) and trial number (MPE: 270 levels; force compensation: 30 levels). Final differences between the experimental groups at the end of exposure were examined using an ANOVA with main effect of experimental group (2 levels) on the kinematic error (last 4 exposure trials) and force compensation (last 4 channel trials). The channel trials (40 per participant) are generated in a pseudorandom fashion. As we are interested in comparing the rate of learning across the two groups we calculate the average of the responses as a function of the trial number such that the occurrence information (the specific trial number of each channel trial) is retained. Details of this averaging procedure

are outlined in the dual-rate state space model description. For plotting purposes only this was smoothed using a ten point running average.

We examined whether the kinematics of the movements to the central target differed between the consistent and variable groups for the same kinematic variables as in Experiment 1. For the pre-exposure trials (null field trials) we calculated the mean and standard deviation (SD) of each measure for each participant and used repeated measures ANOVA to compare the means and SD between the groups.

Dual-rate state space model

We fit a dual-rate state-space model [S8] to the force compensation data for each group. The dual-rate state-space model is described by the following equations:

$$x_f(n+1) = A_f \cdot x_f(n) + B_f \cdot e(n)$$
$$x_s(n+1) = A_s \cdot x_s(n) + B_s \cdot e(n)$$
$$x(n) = x_f(n) + x_s(n)$$
$$e(n) = p(n) - x(n)$$

where x_f(n) and x_s(n) are the states of the fast and slow processes on trial n, x(n) is the total output and e(n) is the error. A_f & A_s are the retention factors for the fast and slow processes and B_f & B_s are the corresponding learning rates. The perturbation p(n) is set to 1 for exposure trials and 0 for null field trials whereas the error e(n) on channel trials is set to 0.

For each of the groups, we fit a single set of parameters (A_f, A_s, B_f, B_s) across the participants [S8]. To do this, for a possible setting of the parameters we simulated the exposure phase for each participant (the simulation depends on the order of the exposure and channel trials which varied across participants). We then averaged the predictions across participants for each trial (which gives the prediction of force

compensation for that trial). We calculated the prediction error for each trial as the squared difference between this prediction and the average of the participants' data who experienced a channel trial on that trial (and weighted this value by the number of participants who experienced a channel trial on that trial). We summed these errors across all exposure trials and set the parameters to minimize this error using fmincon in Matlab.

To generate confidence intervals for our parameter estimates we performed block bootstrapping in which we left out each possible set of three participants from each group and fitted the remaining 9. We used the distribution of parameters across these 220 fits to estimate the confidence limits. To test whether each parameter varied between the two groups we generated all possible differences in each parameter from our bootstrap to generate a new bootstrap sample (220x220 samples).

Experiment 3 – Nonlinear interaction of lead-in and follow-through (n=6).

Each trial could start from one of two possible starting locations (S1 & S2) and end at one of two possible target locations (T1 & T2). Between the start and target locations participants had to pass through initial and final via points (V1 & V2). The start positions were located 10 cm from the initial via point at either 135° or 225°, and targets were located 10 cm from the final via point at either -45° or +45°, giving rise to 4 different possible paths (Fig. 3A). For movement to the initial via point and after the final via point a null field was applied. During the movement between the via points ether a null field, a curl force-field or a force channel could be applied. The curl field coefficient k was set equal to either ±13 N m^{-1} s. Channel trials used a wall that had a spring constant of 10,000 N/m and a perpendicular damping coefficient of 40Nm^{-1} s.

On curl field trials, the field direction (CW or CCW) varied pseudo-randomly from trial to trial. The field direction was determined by the nonlinear XOR rule applied to the starting positions and target position (Fig. 3A). Therefore the field direction could not be predicted based on either the start or target alone but was determined by their combination.

Participants were required to move within 1.25 cm of both via points before reaching the final target and failure to do this led to the trial being aborted. Movement duration was taken as the time between leaving the first via point by 1.25 cm and reaching within 1.25 cm of the second via point. On exposure trials, the curl field was also turned on during this period. If this duration was within 400-700 ms, a “correct speed” message was displayed, otherwise a too fast / too slow warning was given. To avoid initiating the channel before participants were moving straight to the second via point, the channel was applied once the hand was 2 cm away from the center of the first via point.

Blocks consisted of 32 null or field trials and 4 channel trials. Participants performed 5 daily sessions. Day 1 consisted of 216 null trials and 1512 exposure trials, days 2-4 1728 exposure trials and day 5 1944 exposure trials and 72 null trials, giving 8928 trials in total. Participants were given a short rest on average every 200 trials (195-205 trials). Three participants performed the experiments with the fields related to the movements as in Figure 3A whereas the other three participants performed it with the reversed relationship.

Analysis

Differences in kinematic error and force compensation between the first 4 blocks and final 4 blocks in the exposure phase were examined using an ANOVA with a main factor of epoch (2 levels) and random factor of participants (5 levels).

To quantify final learning, we analysed the last 80 force compensation trials for each participant (20 channel trials for each possible movement path). For each participant we averaged the force compensation for each movement path and used multiple linear regression on these values to assess whether the level of force compensation across the four trial types (i.e. S1/T1 S2/T1 S1/T2 S2/T2) associated with the field directions (e.g. CW CCW CCW CW) depended on different features of the trial. We used four different regression contrasts across the trial types to examine the effects of starting

location (+1 -1 +1 -1), final location (+1 +1 -1 -1), the XOR of the starting and final locations (i.e. field direction; +1 -1 -1 +1) and bias (independent of starting and ending location; +1 +1 +1 +1). These four representations form an orthogonal basis set and we can therefore fully partition the force compensation into these four components. The regression analysis is guaranteed to fit the data perfectly (and sums to 100%) as we have 4 measures (the adaptation in the 4 different conditions) and 4 regressors (bias, start, target and XOR). Note that the regression is not about the goodness of fit of the model but is used to determine the extent to which any amount of adaptation seen is determined by the patterns expected for the different contexts. We averaged the variance explained by each basis across the participants.

Relation between the experiments.

Each of the three experiments was designed to test a specific aspect of the effect of follow-through and have very different features, which would lead us a priori to expect different amounts of final learning. For example, Experiments 1 and 3 test the ability to simultaneously learn opposing force fields which is hard whereas in Experiment 2 participants learn only a single force field, which easier. Moreover, each experiment is run for a markedly different number of trials, ranging from 400 (Experiment 2) to 8928 (Experiment 3). Therefore, we do not compare across experiments and all the important comparisons are within each experiment.

Supplemental References

S1. Howard, I. S., Wolpert, D. M., and Franklin, D. W. (2013). The effect of contextual cues on the encoding of motor memories. J Neurophysiol *109*, 2632–2644.

S2. Hwang, E. J., Donchin, O., Smith, M. A., and Shadmehr, R. (2003). A gain-field encoding of limb position and velocity in the internal model of arm dynamics. Plos Biol *1*, E25.

S3. Oldfield, R. C. (1971). The assessment and analysis of handedness: the Edinburgh inventory. Neuropsychologia *9*, 97–113.

S4. Howard, I. S., Ingram, J. N., and Wolpert, D. M. (2009). A modular planar robotic manipulandum with end-point torque control. J. Neurosci. Methods *181*, 199–211.

S5. Gandolfo, F., Mussa-Ivaldi, F. A., and Bizzi, E. (1996). Motor learning by field approximation. Proc. Natl. Acad. Sci. U.S.A. *93*, 3843–3846.

S6. Scheidt, R. A., Reinkensmeyer, D. J., Conditt, M. A., Rymer, W. Z., and Mussa-Ivaldi, F. A. (2000). Persistence of motor adaptation during constrained, multi-joint, arm movements. J Neurophysiol *84*, 853–862.

S7. Milner, T. E., and Franklin, D. W. (2005). Impedance control and internal model use during the initial stage of adaptation to novel dynamics in humans. J. Physiol. (Lond.) *567*, 651–664.

S8. Smith, M. A., Ghazizadeh, A., and Shadmehr, R. (2006). Interacting adaptive processes with different timescales underlie short-term motor learning. Plos Biol *4*, e179.

S9. Howard, I. S., Ingram, J. N., Franklin, D. W., and Wolpert, D. M. (2012). Gone in 0.6 seconds: the encoding of motor memories depends on recent sensorimotor States. Journal of Neuroscience *32*, 12756–12768.
