# Supplementary material for: The Value of the Follow-Through Derives from Motor Learning Depending on Future Actions
Source: Curr Biol. 2015 Feb 2;25(3):397–401. doi: 10.1016/j.cub.2014.12.037 (PMC4320013; doi:10.1016/j.cub.2014.12.037)
Supplement: Document S2. Article plus Supplemental Information [file mmc2.pdf]

Report

Current Biology

The Value of the Follow-Through Derives from Motor Learning Depending on Future Actions

Highlights

- Future movements determine which motor memory is currently active and modifiable
- Skills that otherwise interfere can be learned if each has a unique follow-through
- A single skill is learned faster if its follow-through is consistent
- Selection of motor memories depends on both lead-in and follow-through movements

Authors

Ian S. Howard, Daniel M. Wolpert, David W. Franklin

Correspondence

ian.howard@plymouth.ac.uk (I.S.H.),
dwf25@cam.ac.uk (D.W.F.)

In Brief

For a motor skill to be learned, its motor memory must be stored, protected from interference, and reactivated for modification during practice. Howard et al. demonstrate that future movement determines which motor memory is currently active and that a consistent follow-through constrains skill acquisition to a single memory, thereby speeding up learning.

Howard et al., 2015, Current Biology *25*, 397–401
February 2, 2015 ©2015 Elsevier Ltd All rights reserved
http://dx.doi.org/10.1016/j.cub.2014.12.037

CrossMark

CellPress

Current Biology 25, 397–401, February 2, 2015 ©2015 The Authors http://dx.doi.org/10.1016/j.cub.2014.12.037

Report

The Value of the Follow-Through Derives from Motor Learning Depending on Future Actions

Ian S. Howard,[1,*] Daniel M. Wolpert,[2,3]
and David W. Franklin[2,3,*]
[1]Centre for Robotics and Neural Systems, University of Plymouth, Portland Square, Plymouth PL4 8AA UK
[2]Computational and Biological Learning Lab, Department of Engineering, University of Cambridge, Trumpington Street, Cambridge CB2 1PZ, UK

Summary

In ball sports, we are taught to follow through, despite the inability of events after contact or release to influence the outcome [1, 2]. Here we show that the specific motor memory active at any given moment critically depends on the movement that will be made in the near future. We demonstrate that associating a different follow-through movement with two motor skills that normally interfere [3–7] allows them to be learned simultaneously, suggesting that distinct future actions activate separate motor memories. This implies that when learning a skill, a variable follow-through would activate multiple motor memories across practice, whereas a consistent follow-through would activate a single motor memory, resulting in faster learning. We confirm this prediction and show that such follow-through effects influence adaptation over time periods associated with real-world skill learning. Overall, our results indicate that movements made in the immediate future influence the current active motor memory. This suggests that there is a critical time period both before [8] and after the current movement that determines motor memory activation and controls learning.

Results and Discussion

For a motor skill to be learned over a prolonged period of time, the motor memory of the skill must be stored, protected from interference by intervening tasks, and reactivated for modification when the skill is practiced. Given the widespread notion of the importance of a consistent follow-through in many sports [1, 2], here we examine whether the currently active motor memory might depend on the movement that we are going to make in the near future. We examine a motor skill that is known to be long lasting but also subject to interference—learning to reach in the presence of a dynamic (force-field) perturbation generated on the hand by a robotic interface [4, 9]. When two force fields that act in opposing directions are presented alternately, there is substantial interference, preventing learning of either [4–7]. We first examined whether linking such skills that interfere to different follow-through movements might activate separate motor memories for each, thereby allowing both skills to be learned without interference.

Participants grasped the handle of a robotic interface (Figure S1) and made a reaching movement (in one of four directions) through a perturbing force field to a central target, followed immediately by a second unperturbed, follow-through movement to one of two possible final targets (Figure 1A, follow-through; see the Supplemental Experimental Procedures for full details). The field direction (clockwise or counter-clockwise) was randomly selected on each trial but was uniquely specified by the target (present throughout the trial) to which the follow-through movement would be made (association of force-field direction and follow-through movement was counter-balanced across participants). So that predictive force compensation could be assessed independently from co-contraction, channel trials [10, 11], in which the movement was confined to a simulated mechanical channel from the starting to central target, were randomly applied throughout the experiment. Participants performed 75 blocks of 18 trials each in the force field, and to examine learning, we compared differences in the kinematic error and force compensation between the first four blocks and final four blocks in the exposure phase using an ANOVA with a main factor of epoch (two levels) and random factor of participant (eight levels). We found both a significant reduction in kinematic error (Figure 1B, brown; $F_{1,7} = 16.8$; $p = 0.005$; hand paths shown in Figure S2A) and increase in force compensation (Figure 1C, brown; $F_{1,7} = 17.706$; $p = 0.004$) reaching around 40% of full compensation over a session. In contrast, when a second group of participants were presented with the final target, which again was predictive of the field direction, but did not follow-through to the target (Figure 1A, no follow-through), there was substantial interference between the motor skills, as expected [8, 12, 13]. Although we observed a small reduction in kinematic error in this group (Figure 1B, blue; $F_{1,7} = 12.371$; $p = 0.01$; hand paths shown in Figure S2B) there was no significant increase in force compensation (Figure 1C, blue; $F_{1,7} = 0.434$; $p = 0.531$), suggesting that participants solely used non-specific co-contraction to reduce their error [14–16]. Finally, we contrasted the adaptation in the two groups of subjects using an ANOVA with epoch (two levels) and group (follow-through or no follow-through). There was a significant interaction effect for both kinematic error ($F_{1,124} = 7.388$; $p = 0.08$) and force compensation ($F_{1,124} = 21.55$; $p < 0.001$), indicating that interference was strongly reduced in the follow-through group.

These results show that (despite the unperturbed kinematics of the movements to the central target being similar for both final targets; Table S1), when a follow-through movement is made that is predictive of the field direction, there is substantial reduction in interference. This suggests that different follow-throughs may activate distinct motor memories. Therefore, during skill learning on a single task, identical future movements on each trial (i.e., consistent follow-through) may access a single motor memory. In contrast, a variable follow-through may access multiple motor memories across trials, with any learning being spread across multiple memories, leading to a decrease in the speed of skill acquisition.

We tested this prediction in two groups who experienced a single force field whose direction was fixed across all trials.

[3]Co-senior author
*Correspondence: ian.howard@plymouth.ac.uk (I.S.H.), dwf25@cam.ac.uk (D.W.F.)
This is an open access article under the CC BY license (http://creativecommons.org/licenses/by/4.0/).

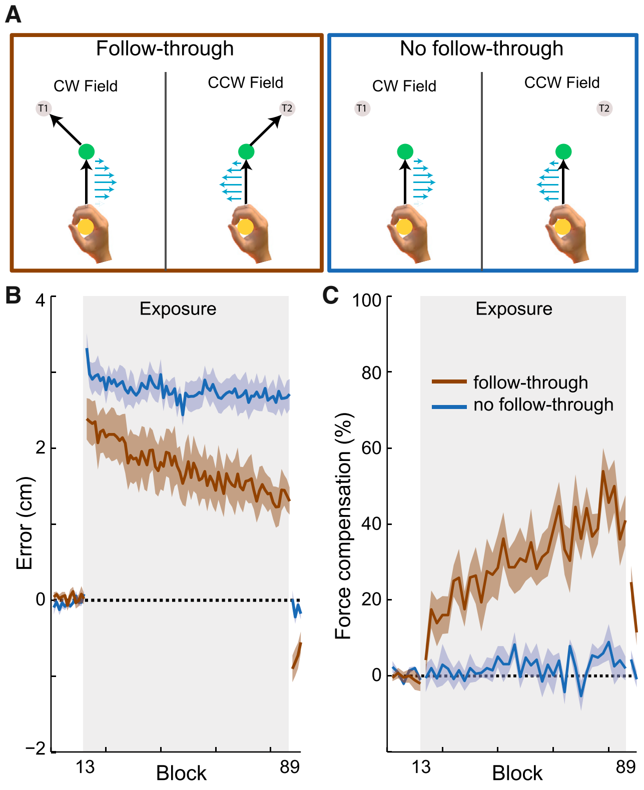

Figure 1. Associating Different Follow-Through Movements with Motor Skills Reduces Interference

(A) Participants made an initial movement to a central target (green circle). During exposure trials, a velocity-dependent curl force field (force vectors shown as blue arrows) was applied during this movement, and the field direction (clockwise [CW] or counter-clockwise [CCW]) was determined by a visual target location (T1 or T2). A follow-through group made a subsequent unperturbed movement to the target location, whereas a no-follow-through group remained at the central target. The directions of the force-field (CW or CCW) and follow-through movement (+45° or −45°) were counter-balanced across participants. Participants made movement in four directions but for clarity only one direction is shown.

(B and C) The kinematic error (B) and force adaptation (C) (mean ± SE across participants for pairs of blocks, combining adjacent even and odd blocks) for the follow-through (brown) and no-follow-through (blue) groups. See also Figures S1 and S2 and Table S1.

Both groups made a movement in the force field to a central target followed by an unperturbed follow-through movement to a final target (Figure 2A). For one group, the follow-through movement was always made to the same target (consistent follow-through), whereas the other group made a follow-through movement on each trial to a randomly selected target from nine possible locations (variable follow-through). The direction of the force-field and follow-through movements was counter-balanced across participants. Importantly, the kinematics (and variability) of unperturbed movements to the central target were not significantly different between the two groups (Table S2), and there was no difference in the initial errors in the force field between the two groups (ANOVA on maximum perpendicular error of the first two exposure trials with a main factor of experimental group: $F_{1,46} = 0.801$; $p = 0.375$). However, significantly faster learning was observed for the consistent, compared to the variable, follow-through group for both kinematic error (Figure 2B; $F_{1,5940} = 155.041$; $p < 0.001$) and force compensation (Figure 2C; $F_{1,660} = 3.921$; $p = 0.048$) as shown using an ANOVA across all exposure trials with the main effects of experimental group and trial number. The same analysis, performed on the first one-third of the exposure trials, showed significantly faster learning for the consistent compared to the variable follow-through group for both kinematic error ($F_{1,1980} = 93.171$; $p < 0.001$) and force compensation ($F_{1,220} = 9.057$; $p = 0.003$). By the end of the session, there were no significant differences in either the kinematic error ($F_{1,94} = 1.668$; $p = 0.2$) or force compensation ($F_{1,94} = 0.163$; $p = 0.687$) (ANOVA on the last four trials with the main effect of experimental group), showing that both groups eventually learned the same amount for this simple skill.

We used a dual-rate model to examine whether changes in the parameters that govern learning might account for the differences that we observed in skill acquisition rate, but not final level of learning. The time course of learning novel dynamics is well accounted for by two interacting processes: a fast process that adapts and decays quickly and a slower process that adapts and decays more gradually [17]. Each process is characterized by a learning rate that controls how strongly the motor memory is updated based on errors and a retention factor determining the movement-to-movement retention of the motor memory. We fit this dual-rate model to our participants' learning (model is fit to the group-averaged data; Figure 2C, thick lines), and this showed that the differences between the groups was primarily due to the retention factor of the fast process (Figure 2D; A_{fast} $p < 0.001$ between groups, other parameters non-significant), suggesting that variable follow-through leads to decreased retention across trials [18].

Could our results on simple force-field learning over the course of an experimental session apply to real-world learning taking place over much more extended periods? In real-world tasks, such as a tennis or golf stroke, the lead-in to the movement is critical for task success, as it will determine characteristics such as variability at contact [19, 20]. Moreover, the recent past has been shown to also affect the selection of the current motor memory [8]. We examined the extent to which two motor skills, opposing force fields, could be simultaneously learned when the skill being currently experienced depended on a nonlinear combination of the past (lead-in) and future (follow-through) movements.

Participants made movements from two possible starting locations (Figure 3A; S1 or S2) through two via points (V1 and V2) to one of two possible target locations (T1 or T2). A force field was applied between the via points whose direction on each trial was uniquely specified, according to an exclusive-or (XOR) rule, by the starting and target locations used on that trial (Figure 3A). Critically, the direction of the force field could not be predicted based on either the start location or the target location alone, but rather depended on both the start and final locations in a non-linear manner. The direction of the force field relative to the movements was counter-balanced across participants. Participants performed 240 blocks of 26 trials over 5 days, and to examine learning, we compared differences in the kinematic error and force compensation for the first four blocks and final four blocks in the force field using an ANOVA with a main factor of epoch (two levels) and random factor of participants (six levels). This was a surprisingly hard task to learn, and over the 5 days of practice, participants showed both a strong and gradual reduction in error (Figure 3B; $F_{1,5} = 36.750$; $p = 0.002$) and increase in force compensation (Figure 3C; $F_{1,5} = 61.981$; $p = 0.001$) to around 50%. Participants learned to access the motor memories based on the nonlinear rule as

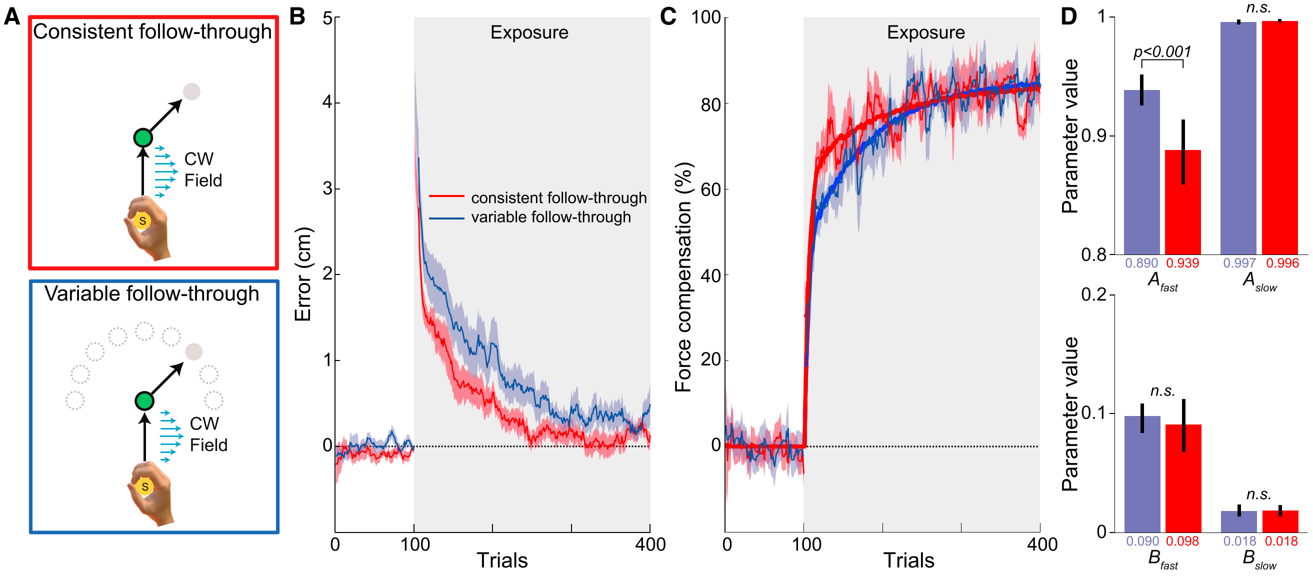

Figure 2. Consistent Follow-Through Improves Learning Rate

(A) Participants made a movement to a central target (green circle) followed by a follow-through movement to a target. During exposure trials, a curl force field was applied on the movement to the central target. The consistent-follow-through group always made the follow-through movement to the same target, whereas for the variable-follow-through group the target was randomly selected from nine possible locations on each trial. The direction of the force-field and follow-through movement was counter-balanced across participants.

(B and C) The kinematic error (B) and force adaptation (C) (ten-trial running mean ± SE across participants) for consistent-follow-through (red) and variable-follow-through (blue) groups. Solid lines show fits of a dual-rate model to force compensation. There are 40 channel trials for each participant, plotted according to the trial number at which they were presented in a pseudo-random fashion.

(D) Parameters of fits (with 95% confidence intervals) of the dual-rate model to both groups.

See also Table S2.

shown by the force compensation being of the appropriate sign for all four possible movements (Figures 3D and S3), with the largest variance in the data accounted for by an XOR rule (Figure 3E). These results demonstrate that participants can utilize both past and future movements to produce a nonlinear separation of motor memories. This shows that the temporal events in close proximity to a movement, both before and after, are critical in determining the motor memory in which the skill is stored. Moreover, even for a relatively simple skill, such as force-field learning (compared to a tennis or golf stroke), the learning is slowly acquired over time courses on the order of real-world skill learning and is highly dependent on both the lead-in and follow-through. Although we have focused on learning simple force fields in constrained arm movements, previous work suggests that the mechanisms underlying such learning generalize to whole-body movement such as posture [21] and walking [22–24], as well as other more naturalistic movements [25].

Previous studies have examined a range of contextual cues that might allow the separation of motor memories. While static cues (e.g., color) have a very limited ability to separate motor memories [12, 13], dynamic moving cues [13, 26], different concurrent motion of the other arm [27–29], or the lead-in to a movement [8, 30] often have a substantial effect. Moreover, separating the location of learning either proprioceptively or visually facilitates learning of opposing force fields [13, 31, 32]. Models of such contextual effects in the motor system posit that they arise from the engagement of separate neural populations, e.g., [27]. Since it known that that future motor planning affects neural activity [33], it seems likely that the follow-through effect that we report either directly engages the separate neural populations that leads to the generation of movement or does so by affecting the initial state of the dynamical systems of neurons in the motor system that control movement [34].

Although we have shown that consistent follow-through leads to faster learning through selection of a single memory, this does not preclude other potential advantages of the follow-though, such as injury reduction or other biomechanical advantages [2]. Although several contextual cues have previously been shown to reduce interference [13, 26, 27, 29, 30, 32, 35, 36], our study is the first to show that different follow-through movements can reduce interference substantially, demonstrating the importance of future motor events in controlling current motor learning. Our findings suggest that distinct follow-throughs associated with different motor skills, such as different tennis strokes, will help maintain these skills in separate motor memories, thereby protecting them from interference when learning other skills. Moreover, even for a single skill, maintaining a consistent follow-through will speed up learning. An intriguing question is why a particular follow-through might be preferred when learning a skill. Our results suggest that variability in the follow-through, which might arise from planning variability [37], motor noise [19, 20, 38], or other sources of variability [39], would lead to a reduction in the speed of skill acquisition. Therefore, it may be optimal to choose the follow-through for a skill that can be executed with the minimum variability.

Supplemental Information

Supplemental Information includes Supplemental Experimental Procedures, three figures, and two tables and can be found with this article online at http://dx.doi.org/10.1016/j.cub.2014.12.037.

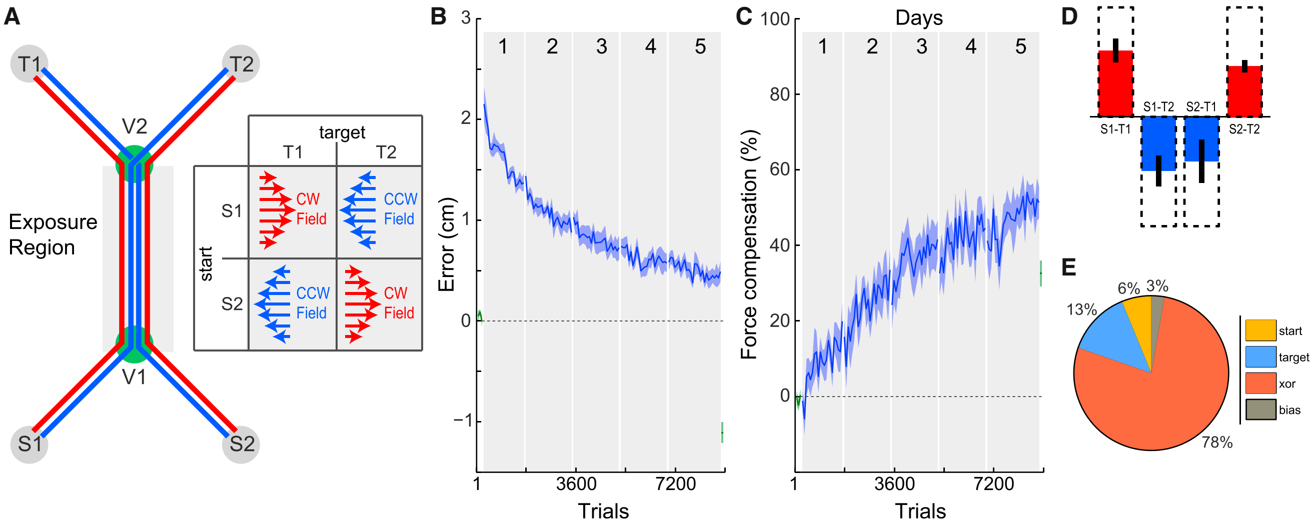

Figure 3. Participants Show Extended Learning of Skills that Nonlineary Depend on Both Lead-In and Follow-Through

(A) Participants made movements from one of two start locations to one of two target locations (four possible movements). Movements had to pass through two via points (V1 and V2). On each exposure trial, a force field was applied in the region between the via points (exposure region), and the field direction (CW or CCW) was uniquely specified by the combination of the start and target locations according to an XOR rule (table). The direction of the force field (CW or CCW) was counter-balanced across participants.
(B and C) The kinematic error (B) and force adaptation (C) (mean ± SE across participants for pairs of blocks, combining adjacent even and odd blocks) over the 5 days of the experiment.
(D) The force compensation averaged over the last 20 blocks (mean ± SE across participants) for each of the four movements, with dashed bars indicating full compensation.
(E) Average of the variance explained by a regression analysis across participants. The regression analysis was used to predict the pattern of compensation (D) by fitting weights to the four possible patterns (bias, start, target, XOR) across the movements. The percentage shows the amount of the variance in force compensation explained by these four patterns (see the Supplemental Experimental Procedures for details).
See also Figure S3.

Author Contributions

I.S.H., D.M.W., and D.W.F. designed the study. I.S.H. ran the experiments. I.S.H., D.M.W., and D.W.F. performed the data analysis. I.S.H., D.M.W., and D.W.F. wrote the paper.

Acknowledgments

We thank the Wellcome Trust (WT091547MA and WT097803MA), Human Frontier Science Program, Plymouth University, and Royal Society (Noreen Murray Professorship in Neurobiology to D.M.W.) for support. We thank Sae Franklin and Christopher Ford for their assistance with running the experiments and Amy Bastian for comments on the manuscript.

Received: September 8, 2014
Revised: October 28, 2014
Accepted: December 15, 2014
Published: January 8, 2015

References

1. Karageanes, S.J., and Scher, S. (2005). Baseball. In Principles of Manual Sports Medicine, S.J. Karageanes, ed. (Lippincott Williams & Wilkins), pp. 453–464.
2. Knudson, D., and Elliott, B. (2004). Biomechanics of tennis stroke. In Biomedical Engineering Principles in Sports, G.K. Hung and J.M. Pallis, eds. (Kluwer Academic), pp. 153–181.
3. Shadmehr, R., and Holcomb, H.H. (1997). Neural correlates of motor memory consolidation. Science *277*, 821–825.
4. Brashers-Krug, T., Shadmehr, R., and Bizzi, E. (1996). Consolidation in human motor memory. Nature *382*, 252–255.
5. Caithness, G., Osu, R., Bays, P., Chase, H., Klassen, J., Kawato, M., Wolpert, D.M., and Flanagan, J.R. (2004). Failure to consolidate the consolidation theory of learning for sensorimotor adaptation tasks. J. Neurosci. *24*, 8662–8671.
6. Karniel, A., and Mussa-Ivaldi, F.A. (2002). Does the motor control system use multiple models and context switching to cope with a variable environment? Exp. Brain Res. *143*, 520–524.
7. Krakauer, J.W., Ghilardi, M.F., and Ghez, C. (1999). Independent learning of internal models for kinematic and dynamic control of reaching. Nat. Neurosci. *2*, 1026–1031.
8. Howard, I.S., Ingram, J.N., Franklin, D.W., and Wolpert, D.M. (2012). Gone in 0.6 seconds: the encoding of motor memories depends on recent sensorimotor states. J. Neurosci. *32*, 12756–12768.
9. Shadmehr, R., and Brashers-Krug, T. (1997). Functional stages in the formation of human long-term motor memory. J. Neurosci. *17*, 409–419.
10. Scheidt, R.A., Reinkensmeyer, D.J., Conditt, M.A., Rymer, W.Z., and Mussa-Ivaldi, F.A. (2000). Persistence of motor adaptation during constrained, multi-joint, arm movements. J. Neurophysiol. *84*, 853–862.
11. Milner, T.E., and Franklin, D.W. (2005). Impedance control and internal model use during the initial stage of adaptation to novel dynamics in humans. J. Physiol. *567*, 651–664.
12. Gandolfo, F., Mussa-Ivaldi, F.A., and Bizzi, E. (1996). Motor learning by field approximation. Proc. Natl. Acad. Sci. USA *93*, 3843–3846.
13. Howard, I.S., Wolpert, D.M., and Franklin, D.W. (2013). The effect of contextual cues on the encoding of motor memories. J. Neurophysiol. *109*, 2632–2644.
14. Burdet, E., Osu, R., Franklin, D.W., Milner, T.E., and Kawato, M. (2001). The central nervous system stabilizes unstable dynamics by learning optimal impedance. Nature *414*, 446–449.
15. Osu, R., Franklin, D.W., Kato, H., Gomi, H., Domen, K., Yoshioka, T., and Kawato, M. (2002). Short- and long-term changes in joint co-contraction associated with motor learning as revealed from surface EMG. J. Neurophysiol. *88*, 991–1004.
16. Franklin, D.W., Osu, R., Burdet, E., Kawato, M., and Milner, T.E. (2003). Adaptation to stable and unstable dynamics achieved by combined impedance control and inverse dynamics model. J. Neurophysiol. *90*, 3270–3282.
17. Smith, M.A., Ghazizadeh, A., and Shadmehr, R. (2006). Interacting adaptive processes with different timescales underlie short-term motor learning. PLoS Biol. *4*, e179.

18. Ingram, J.N., Flanagan, J.R., and Wolpert, D.M. (2013). Context-dependent decay of motor memories during skill acquisition. Curr. Biol. *23*, 1107–1112.
19. Harris, C.M., and Wolpert, D.M. (1998). Signal-dependent noise determines motor planning. Nature *394*, 780–784.
20. Todorov, E., and Jordan, M.I. (2002). Optimal feedback control as a theory of motor coordination. Nat. Neurosci. *5*, 1226–1235.
21. Ahmed, A.A., and Wolpert, D.M. (2009). Transfer of dynamic learning across postures. J. Neurophysiol. *102*, 2816–2824.
22. Emken, J.L., Benitez, R., Sideris, A., Bobrow, J.E., and Reinkensmeyer, D.J. (2007). Motor adaptation as a greedy optimization of error and effort. J. Neurophysiol. *97*, 3997–4006.
23. Finley, J.M., Bastian, A.J., and Gottschall, J.S. (2013). Learning to be economical: the energy cost of walking tracks motor adaptation. J. Physiol. *591*, 1081–1095.
24. Torres-Oviedo, G., and Bastian, A.J. (2012). Natural error patterns enable transfer of motor learning to novel contexts. J. Neurophysiol. *107*, 346–356.
25. Ingram, J.N., and Wolpert, D.M. (2011). Naturalistic approaches to sensorimotor control. Prog. Brain Res. *191*, 3–29.
26. Cothros, N., Wong, J., and Gribble, P.L. (2009). Visual cues signaling object grasp reduce interference in motor learning. J. Neurophysiol. *102*, 2112–2120.
27. Nozaki, D., Kurtzer, I., and Scott, S.H. (2006). Limited transfer of learning between unimanual and bimanual skills within the same limb. Nat. Neurosci. *9*, 1364–1366.
28. Howard, I.S., Ingram, J.N., and Wolpert, D.M. (2010). Context-dependent partitioning of motor learning in bimanual movements. J. Neurophysiol. *104*, 2082–2091.
29. Yokoi, A., Hirashima, M., and Nozaki, D. (2011). Gain field encoding of the kinematics of both arms in the internal model enables flexible bimanual action. J. Neurosci. *31*, 17058–17068.
30. Wainscott, S.K., Donchin, O., and Shadmehr, R. (2005). Internal models and contextual cues: encoding serial order and direction of movement. J. Neurophysiol. *93*, 786–800.
31. Hwang, E.J., Smith, M.A., and Shadmehr, R. (2006). Dissociable effects of the implicit and explicit memory systems on learning control of reaching. Exp. Brain Res. *173*, 425–437.
32. Hirashima, M., and Nozaki, D. (2012). Distinct motor plans form and retrieve distinct motor memories for physically identical movements. Curr. Biol. *22*, 432–436.
33. Cisek, P., and Kalaska, J.F. (2005). Neural correlates of reaching decisions in dorsal premotor cortex: specification of multiple direction choices and final selection of action. Neuron *45*, 801–814.
34. Churchland, M.M., Cunningham, J.P., Kaufman, M.T., Foster, J.D., Nuyujukian, P., Ryu, S.I., and Shenoy, K.V. (2012). Neural population dynamics during reaching. Nature *487*, 51–56.
35. Addou, T., Krouchev, N., and Kalaska, J.F. (2011). Colored context cues can facilitate the ability to learn and to switch between multiple dynamical force fields. J. Neurophysiol. *106*, 163–183.
36. Imamizu, H., Sugimoto, N., Osu, R., Tsutsui, K., Sugiyama, K., Wada, Y., and Kawato, M. (2007). Explicit contextual information selectively contributes to predictive switching of internal models. Exp. Brain Res. *181*, 395–408.
37. Churchland, M.M., Afshar, A., and Shenoy, K.V. (2006). A central source of movement variability. Neuron *52*, 1085–1096.
38. van Beers, R.J. (2009). Motor learning is optimally tuned to the properties of motor noise. Neuron *63*, 406–417.
39. Beck, J.M., Ma, W.J., Pitkow, X., Latham, P.E., and Pouget, A. (2012). Not noisy, just wrong: the role of suboptimal inference in behavioral variability. Neuron *74*, 30–39.

Current Biology
Supplemental Information

The Value of the Follow-Through Derives from Motor Learning Depending on Future Actions

Ian S. Howard, Daniel M. Wolpert, and David W. Franklin

Supplemental Figures

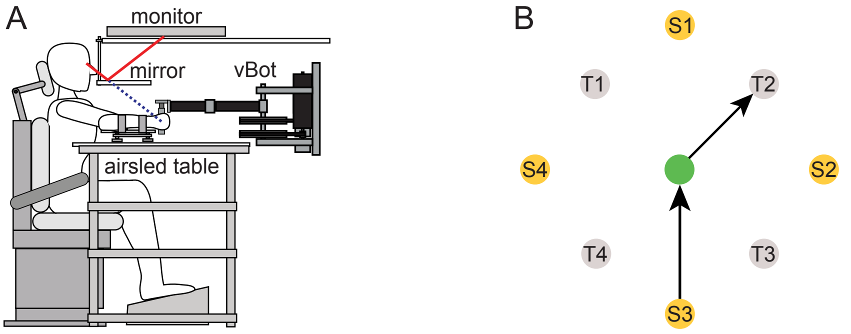

Figure S1. Experimental Paradigm.

(A) The participant grasps the handle of the robotic manipulandum (vBOT) while seated. Visual feedback of movements is presented veridically using a horizontally mounted monitor viewed through a mirror. The participant's forearm is fixed to the handle and supported by an airsled.

(B) Workspace layout for Experiment 1. There were four possible final target locations (grey circles: T1-T4), one central target (green circle, note that in the experiment this was displayed as grey) and four start target locations (yellow circles: S1-S4). For each start location, two possible final target locations could be chosen (one for each force-field direction) corresponding to ±45° angle relative to the initial movement direction. The arrows show an example of the movements in a single trial for the follow-through group.

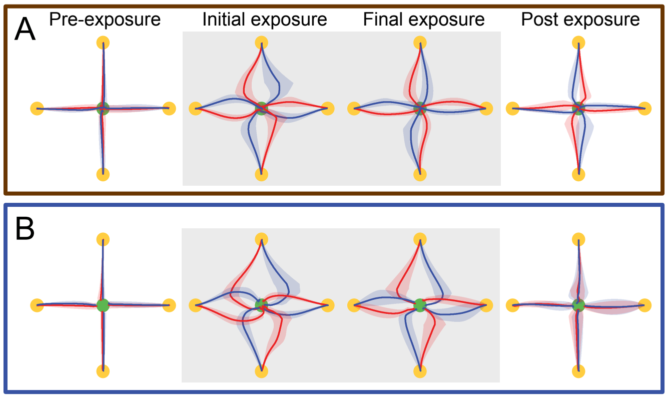

Figure S2. Hand paths for the four different movements to the central target for Experiment 1.

(A) Hand paths (mean ± s.d. across participants) to the central target during different phases on the experiment for the follow-through group; pre-exposure (last block), initial exposure (first block), final exposure (last block), post exposure (first block). Each trajectory is the average of the first trajectory from each participant within each relevant block.

(B) Hand paths to the central target for the no follow-through group.

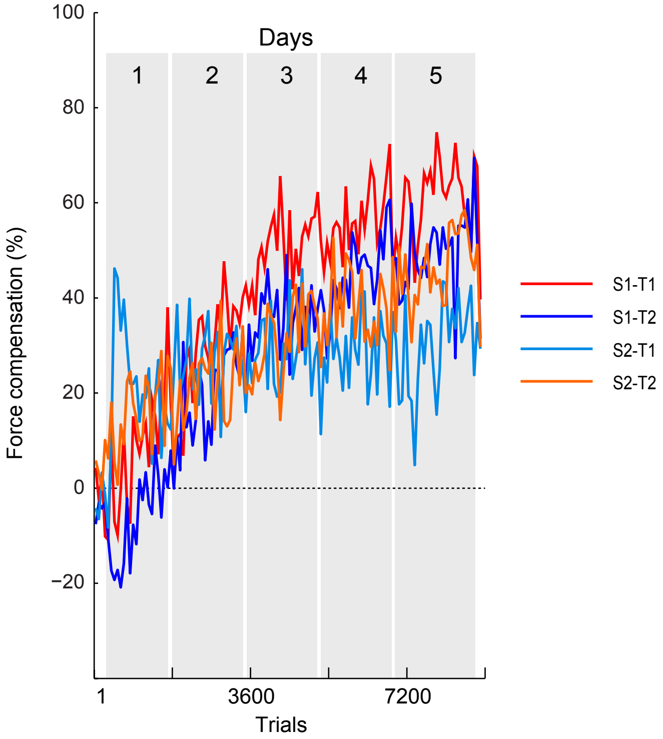

Figure S3. Evolution of force compensation for each movement in Experiment 3.

The force adaptation (mean across participants for pairs of blocks) over the 5 days of the experiment for each of the four movements.

Supplemental Tables

	No follow-through group				Follow-through group				Group difference		
	Target				Target						
Measure	Left	Right	$F_{1,7}$	p	Left	Right	$F_{1,7}$	p	Difference	$F_{1,14}$	p
Lateral deviation (cm)	-0.02	-0.04	0.41	0.542	0.01	-0.08	5.88	0.046	0.00	0.000	0.988
Path length (cm)	9.7	9.8	1.87	0.214	9.7	9.7	0.45	0.522	-0.03	0.65347	0.432
Duration (s)	0.272	0.273	0.04	0.852	0.235	0.237	0.84	0.390	-0.036	6.3768	0.024
Peak speed (cm/s)	46.0	46.1	0.07	0.796	52.9	52.7	0.66	0.444	6.7	4.0337	0.064
Central target speed (cm/s)	27.4	27.2	0.24	0.642	33.5	32.9	2.10	0.191	5.9	5.8287	0.030
Dwell time (s)					0.146	0.151	1.45	0.268			

Table S1. Analysis of the kinematics for the pre-exposure (null field) trials of Experiment 1.

For each group we performed a repeated measures ANOVA for each kinematic measure as a factor of final target direction (2 levels). Table shows means for each group, the F statistics and p-values comparing left and right final targets (i.e. different follow-troughs or visual targets). We also examined the differences between the groups using a repeated measures ANOVA with a single factor of group. The table shows the mean difference between the groups, F statistics and p values. Note that, with one exception, the differences between the two targets are not significant. The only significant difference is the lateral deviation at mid-movement for which the mean difference is 0.9 mm and below the spatial difference that allows opposing fields to be learned [S1, S2]. Not surprisingly, there are differences in the kinematic parameters and duration and central target velocity for the follow-through compared to no follow-through groups, as the latter were required to stop at the central target. Other kinematic parameters were not significantly different.

	Kinematic measures				Kinematic variability (SD)			
	Group		Difference		Group		Difference	
**Measure**	**Consistent**	**Variable**	**$F_{1,22}$**	**p**	**Consistent**	**Variable**	**$F_{1,22}$**	**p**
Lateral deviation (cm)	-0.019	-0.095	0.81	0.38	0.41	0.44	0.35	0.56
Path length (cm)	15.7	15.7	0.89	0.35	0.45	0.41	0.07	0.79
Duration (s)	0.34	0.34	0.26	0.62	0.077	0.084	0.04	0.84
Peak speed (cm/s)	63.3	64.7	0.11	0.74	8.1	8.3	0.03	0.86
Central target speed (cm/s)	28.6	28.2	0.06	0.81	10.5	10.6	0.01	0.92
Dwell time (s)	0.291	0.269	1.27	0.27	0.079	0.074	0.18	0.68

Table S2. Analysis of the kinematics for the pre-exposure (null field) trials of Experiment 2.

For each kinematic measure we examined whether the two groups (variable and consistent follow-through) differed in their mean values and in their variability (as reflected in the SD of the kinematic measured calculated within each participant). We used repeated measure ANOVAs with a single factor of group. The table shows the mean and the average SD for each group and the F statistics and p values testing for the difference between the groups. All tests showed the differences failed to reach significance ($p>0.27$ for all).

Supplemental Experimental Procedures

A total of 46 right-handed participants (16 female; age 22.7±4.9 mean±sd years) took part in the experiments. Participants provided written informed consent and were naïve to the aims of the experiments. Local ethics committees in Cambridge and in Plymouth approved the protocol and all participants were right handed based on the Edinburgh handedness questionnaire [S3].

Apparatus

Experiments were performed using a vBOT planar robotic manipulandum, with associated virtual reality system and air table (for details see [S4]). Participants were seated in a sturdy chair in front of the apparatus and firmly strapped against the backrest with a four-point seatbelt to reduce body movement. Participants grasped the robot handle in their right hand while an air sled (constraining movement to the horizontal plane) supported their right forearm. Handle position and endpoint force (Nano 25 6-axis transducer; ATI) were recorded at 1000 Hz for offline analysis using Matlab (Matlab, The MathWorks Inc., Natick, MA, USA).

Visual feedback was provided using a computer monitor mounted above the vBOT and was projected veridically to the participant via a mirror. The virtual reality system was used to overlay images such as targets (1.25 cm radius disks) and a hand cursor (0.5 cm radius red disk) in the plane of movement.

Experiment 1 – The effect of follow-through movements on interference (n=16).

Experiment 1 was designed to examine the ability of future movements to separate current motor memories. On each trial a starting, central and final target was displayed. The central target was in a fixed position on all trials, approximately 30 cm below the eyes and 30 cm in front of the chest. There were four possible starting targets positioned 12 cm from the central target and arranged at 0°, 90°, 180° and 270°.

The final target was 10 cm from the central target and located either at -45° or +45° relative to the starting-to-central target direction (Fig. S1).

At the beginning of a trial, the starting location was displayed to which the vBOT moved the participant's hand (following a minimum jerk trajectory), at which point the central and a final target were displayed. Participants were required to remain within the start target for 300 ms, after which they were cued by a tone to initiate the movement. Participants were required to make a movement to the central target during which either a null field, a velocity-dependent curl force-field [S5] or a force channel was presented. In null field trials, the vBOT generated no force. In the curl force-field trials, the force generated by the vBOT was given by:

$$\begin{bmatrix} F_x \\ F_y \end{bmatrix} = k \begin{bmatrix} 0 & -1 \\ 1 & 0 \end{bmatrix} \begin{bmatrix} \dot{x} \\ \dot{y} \end{bmatrix}$$

where *k* was set equal to ±13 N m^{-1} s. The sign of *k* determined the direction of the force-field (clockwise CW or counterclockwise CCW). The direction of the force-field was determined by the whether the final target was displayed at +45° or -45° relative to the movement to the central target (e.g. +45=CW & -45°=CCW). The assignment between direction to the final target (+45/-45°) and curl field direction (CW/CCW) was fixed within a participant but counterbalanced across participants.

Channel trials were used to assess feedforward adaptation. In a channel trial, the movement was confined to a simulated mechanical channel from the starting to central target with a spring constant of 10,000 N/m and a damping coefficient of 50 $Nm^{-1}s$ orthogonal to the wall [S6, S7].

A block consisted of 16 field trials and 2 channel trials. In the field trials, each of the 4 starting targets and 2 possible targets was repeated twice. The order of the trials within a block was pseudo-random, except that a channel trial always occurred within the first and last four trials of each block. Channel trials were

only applied for movements from the 0° start position, with one trial for each of the two possible final targets (randomizing which came first within each block).

Each experiment began with a pre-exposure phase consisting of 12 blocks in which no forces were applied (216 null trials), followed by an exposure phase of 75 blocks (1350 field trials), and finally a post-exposure phase consisting of 4 blocks (72 null trials). Participants were given a short rest on average every 200 trials (195-205 trials).

Participants were allocated to one of two groups: a no follow-through (n=8) and a follow-through group (n=8), that differed only in events after they had reached through the force-field to the central target. The no follow-through group was required to stop within the central target after which the trial ended. The follow-through group was required to continue the movement from the central target to the final target and they were not required to stop at the central target. If they did not pass through the central target, the trial was terminated. Forces generated by the vBOT were turned off after participants entered the central target, so that movements from the central target to the final target were always made in a null field.

After each trial, participants were given feedback on the duration of their movement to the central target. This was the time from when the hand exceeded a speed of 30 cm/s after it had left the start target until it entered the central target (no follow-through group) or passed the middle of the central target (follow-through group). If the duration was between 150-250 ms, a “correct speed” message was displayed, otherwise a too fast / too slow warning was given. Participants were required to repeat a trial if they failed to achieve a speed greater than 30 cm/s in the movement to the central target.

Analysis

The kinematic error was calculated on each movement to the central target as the maximum perpendicular error (MPE) of the hand path relative to a straight line joining the movement’s start and the central target. For each participant, the MPE for all trials was averaged within a block, with the sign

appropriately reversed so that errors from CW and CCW field trials could be combined. The MPE mean and standard error (SE) was computed across all participants. For plotting, the mean and SE was calculated for each adjacent pair of blocks, combining odd and even blocks (blocks 1 & 2, 3 & 4 etc).

To assess feedforward learning independent of co-contraction we analyzed the force produced on channel trials [S7, S8]. The force produced by participants orthogonal to the channel wall was summed across the movement to the central target. This value was expressed as a percentage adaptation (termed Force Compensation) by calculating the value that would perfectly compensate for the field, as determined by the field strength and the summed movement velocity on that trial [S1, S9].

We also examined whether the kinematics of the movements to the central target depended on whether the final target was at -45° (leftward) or +45° (rightward) of the initial movement. If unperturbed movements to the central target are substantially different for the two possible final targets this could facilitate learning [S1, S2]. We therefore examined the pre-exposure trials (null field trials) as these provide a fair comparison as we expect substantial differences during field trials (as the field direction are different for the different final targets). We calculated 6 kinematic measures for each movement to the central target. We selected the part of the movement from when the hand left the starting location to when it entered the central target and calculated the duration, path length and peak speed of this movement, as well as the final speed as the hand entered the central target. We also calculated the signed lateral deviation from the straight line joining the starting and central target when the hand was midway to the central target. In addition, for the follow-through group we calculated the dwell time that the hand spent within the central target. Within each group we compared these measures for the leftward and rightward targets and also compared these measures across the two groups (collapsed across targets). For each group we performed a repeated measures ANOVA for each kinematic measure as a factor of final target direction (2 levels). Here, multiple ANOVAs are more appropriate than a single MANOVA, as we wish to reduce the chances of a type II error.

We performed hypothesis-based planned comparisons and report uncorrected p-values to determine statistical significance. Statistical differences were determined using an ANOVA in SPSS using the general linear model. Differences in kinematic error or force compensation between the first 4 blocks and final 4 blocks in the exposure phase were examined using an ANOVA with a main factor of epoch (2 levels) and random factor of participants (8 levels). To contrast the adaptation in the two groups an ANOVA was performed with main factors of epoch (2 levels) and experimental group (2 levels: follow-through and no follow-through) to examine whether the interaction between epoch and group was significantly different. Statistical significance was considered at the $p < 0.05$ level for all statistical tests.

Experiment 2 – The effect of follow-through variability on learning (n=24).

We examined how follow-though variability affected the rate of learning of a single force-field. The experiment was similar to first experiment, except that only a single starting location (0°) and single force-field (field constant k increased to 16 N m^{-1} s) was experienced by each participant (Fig. 2A). The field direction (CW or CCW) was counterbalanced across participants, but each participant only experienced one force-field.

Participants were allocated to one of two groups: a consistent follow-through (n=12) and a variable follow-through group (n=12) that differed only in the movement they were required to make after they had reached through the field to the central target (located 18 cm from the start location). The consistent follow-through group always reached to the same final target (counterbalanced at +45° or -45° across participants) whereas for the variable follow-through group the final target was selected on each from one of 9 possible locations. The set of possible final targets were 10 cm from the central target, and arranged between -90° to +90° in 22.5° steps (Fig. 2A). To allow comparison with the consistent follow-through group, for half the participants channel trials occurred only for the +45° final target and for the other half for the -45° target.

Participants were encouraged to pause briefly at the central target on the way to the final target. If they remained within the central target for less than 50 ms a warning was given at the end of the trial. This small pause within the central target was required so as to maintain similar durations and speeds for the movement to the central target for all follow-through directions. The duration of the dwell time was kept small (50 ms) as the strength of the follow-through effect is likely to decay as dwell time increases [S9]. Movement duration was calculated as the time between when the hand left the starting location until it was within the central target. If the duration was within 200-300ms, “great” was displayed, otherwise if within 150-350ms, a “good” was displayed. Outside this range, a too fast / too slow warning was given.

A block consisted of 9 null/field trials and 1 channel trial. Channel trials used a spring constant of 6,000 N/m and a damping field constant of 30 Nm^{-1} s. Each experiment began with a pre-exposure phase consisting of 10 blocks in which no forces were applied (100 null field trials), followed by an exposure phase of 30 blocks (300 field trials), and finally a post-exposure phase consisting of 10 block (100 null trials). There were 40 channel trials in total (10 in the pre-exposure and 30 during the exposure phase).

Analysis

Initial differences in kinematic error upon introduction of the force-field was examined using the MPE on the first 2 exposure trials with an ANOVA with a main factor of experimental group (2 levels). The speed of learning was examined for both kinematic error and force compensation using an ANOVA across all of the exposure trials and over the first third of the exposure trials. The ANOVA was performed with a main effect of experimental group (2 levels) and trial number (MPE: 270 levels; force compensation: 30 levels). Final differences between the experimental groups at the end of exposure were examined using an ANOVA with main effect of experimental group (2 levels) on the kinematic error (last 4 exposure trials) and force compensation (last 4 channel trials). The channel trials (40 per participant) are generated in a pseudorandom fashion. As we are interested in comparing the rate of learning across the two groups we calculate the average of the responses as a function of the trial number such that the occurrence information (the specific trial number of each channel trial) is retained. Details of this averaging procedure

are outlined in the dual-rate state space model description. For plotting purposes only this was smoothed using a ten point running average.

We examined whether the kinematics of the movements to the central target differed between the consistent and variable groups for the same kinematic variables as in Experiment 1. For the pre-exposure trials (null field trials) we calculated the mean and standard deviation (SD) of each measure for each participant and used repeated measures ANOVA to compare the means and SD between the groups.

Dual-rate state space model

We fit a dual-rate state-space model [S8] to the force compensation data for each group. The dual-rate state-space model is described by the following equations:

$$
\begin{aligned}
x_f(n+1) &= A_f \cdot x_f(n) + B_f \cdot e(n) \\
x_s(n+1) &= A_s \cdot x_s(n) + B_s \cdot e(n) \\
x(n) &= x_f(n) + x_s(n) \\
e(n) &= p(n) - x(n)
\end{aligned}
$$

where x_f(n) and x_s(n) are the states of the fast and slow processes on trial n, x(n) is the total output and e(n) is the error. A_f & A_s are the retention factors for the fast and slow processes and B_f & B_s are the corresponding learning rates. The perturbation p(n) is set to 1 for exposure trials and 0 for null field trials whereas the error e(n) on channel trials is set to 0.

For each of the groups, we fit a single set of parameters (A_f, A_s, B_f, B_s) across the participants [S8]. To do this, for a possible setting of the parameters we simulated the exposure phase for each participant (the simulation depends on the order of the exposure and channel trials which varied across participants). We then averaged the predictions across participants for each trial (which gives the prediction of force

compensation for that trial). We calculated the prediction error for each trial as the squared difference between this prediction and the average of the participants' data who experienced a channel trial on that trial (and weighted this value by the number of participants who experienced a channel trial on that trial). We summed these errors across all exposure trials and set the parameters to minimize this error using fmincon in Matlab.

To generate confidence intervals for our parameter estimates we performed block bootstrapping in which we left out each possible set of three participants from each group and fitted the remaining 9. We used the distribution of parameters across these 220 fits to estimate the confidence limits. To test whether each parameter varied between the two groups we generated all possible differences in each parameter from our bootstrap to generate a new bootstrap sample (220x220 samples).

Experiment 3 – Nonlinear interaction of lead-in and follow-through (n=6).

Each trial could start from one of two possible starting locations (S1 & S2) and end at one of two possible target locations (T1 & T2). Between the start and target locations participants had to pass through initial and final via points (V1 & V2). The start positions were located 10 cm from the initial via point at either 135° or 225°, and targets were located 10 cm from the final via point at either -45° or +45°, giving rise to 4 different possible paths (Fig. 3A). For movement to the initial via point and after the final via point a null field was applied. During the movement between the via points ether a null field, a curl force-field or a force channel could be applied. The curl field coefficient k was set equal to either ±13 N m^{-1} s. Channel trials used a wall that had a spring constant of 10,000 N/m and a perpendicular damping coefficient of 40Nm^{-1} s.

On curl field trials, the field direction (CW or CCW) varied pseudo-randomly from trial to trial. The field direction was determined by the nonlinear XOR rule applied to the starting positions and target position (Fig. 3A). Therefore the field direction could not be predicted based on either the start or target alone but was determined by their combination.

Participants were required to move within 1.25 cm of both via points before reaching the final target and failure to do this led to the trial being aborted. Movement duration was taken as the time between leaving the first via point by 1.25 cm and reaching within 1.25 cm of the second via point. On exposure trials, the curl field was also turned on during this period. If this duration was within 400-700 ms, a “correct speed” message was displayed, otherwise a too fast / too slow warning was given. To avoid initiating the channel before participants were moving straight to the second via point, the channel was applied once the hand was 2 cm away from the center of the first via point.

Blocks consisted of 32 null or field trials and 4 channel trials. Participants performed 5 daily sessions. Day 1 consisted of 216 null trials and 1512 exposure trials, days 2-4 1728 exposure trials and day 5 1944 exposure trials and 72 null trials, giving 8928 trials in total. Participants were given a short rest on average every 200 trials (195-205 trials). Three participants performed the experiments with the fields related to the movements as in Figure 3A whereas the other three participants performed it with the reversed relationship.

Analysis

Differences in kinematic error and force compensation between the first 4 blocks and final 4 blocks in the exposure phase were examined using an ANOVA with a main factor of epoch (2 levels) and random factor of participants (5 levels).

To quantify final learning, we analysed the last 80 force compensation trials for each participant (20 channel trials for each possible movement path). For each participant we averaged the force compensation for each movement path and used multiple linear regression on these values to assess whether the level of force compensation across the four trial types (i.e. S1/T1 S2/T1 S1/T2 S2/T2) associated with the field directions (e.g. CW CCW CCW CW) depended on different features of the trial. We used four different regression contrasts across the trial types to examine the effects of starting

location (+1 -1 +1 -1), final location (+1 +1 -1 -1), the XOR of the starting and final locations (i.e. field direction; +1 -1 -1 +1) and bias (independent of starting and ending location; +1 +1 +1 +1). These four representations form an orthogonal basis set and we can therefore fully partition the force compensation into these four components. The regression analysis is guaranteed to fit the data perfectly (and sums to 100%) as we have 4 measures (the adaptation in the 4 different conditions) and 4 regressors (bias, start, target and XOR). Note that the regression is not about the goodness of fit of the model but is used to determine the extent to which any amount of adaptation seen is determined by the patterns expected for the different contexts. We averaged the variance explained by each basis across the participants.

Relation between the experiments.

Each of the three experiments was designed to test a specific aspect of the effect of follow-through and have very different features, which would lead us a priori to expect different amounts of final learning. For example, Experiments 1 and 3 test the ability to simultaneously learn opposing force fields which is hard whereas in Experiment 2 participants learn only a single force field, which easier. Moreover, each experiment is run for a markedly different number of trials, ranging from 400 (Experiment 2) to 8928 (Experiment 3). Therefore, we do not compare across experiments and all the important comparisons are within each experiment.

Supplemental References

S1. Howard, I. S., Wolpert, D. M., and Franklin, D. W. (2013). The effect of contextual cues on the encoding of motor memories. J Neurophysiol *109*, 2632–2644.

S2. Hwang, E. J., Donchin, O., Smith, M. A., and Shadmehr, R. (2003). A gain-field encoding of limb position and velocity in the internal model of arm dynamics. Plos Biol *1*, E25.

S3. Oldfield, R. C. (1971). The assessment and analysis of handedness: the Edinburgh inventory. Neuropsychologia *9*, 97–113.

S4. Howard, I. S., Ingram, J. N., and Wolpert, D. M. (2009). A modular planar robotic manipulandum with end-point torque control. J. Neurosci. Methods *181*, 199–211.

S5. Gandolfo, F., Mussa-Ivaldi, F. A., and Bizzi, E. (1996). Motor learning by field approximation. Proc. Natl. Acad. Sci. U.S.A. *93*, 3843–3846.

S6. Scheidt, R. A., Reinkensmeyer, D. J., Conditt, M. A., Rymer, W. Z., and Mussa-Ivaldi, F. A. (2000). Persistence of motor adaptation during constrained, multi-joint, arm movements. J Neurophysiol *84*, 853–862.

S7. Milner, T. E., and Franklin, D. W. (2005). Impedance control and internal model use during the initial stage of adaptation to novel dynamics in humans. J. Physiol. (Lond.) *567*, 651–664.

S8. Smith, M. A., Ghazizadeh, A., and Shadmehr, R. (2006). Interacting adaptive processes with different timescales underlie short-term motor learning. Plos Biol *4*, e179.

S9. Howard, I. S., Ingram, J. N., Franklin, D. W., and Wolpert, D. M. (2012). Gone in 0.6 seconds: the encoding of motor memories depends on recent sensorimotor States. Journal of Neuroscience *32*, 12756–12768.
